# TFRC and ACTB as the best reference genes to quantify Urokinase Plasminogen Activator in breast cancer

**DOI:** 10.1186/1756-0500-4-215

**Published:** 2011-06-25

**Authors:** Keivan Majidzadeh-A, Rezvan Esmaeili, Nasrin Abdoli

**Affiliations:** 1 Iranian Center for Breast Cancer (ICBC); Academic Center for Education, Culture and Research (ACECR) No 45, Nazari St, Aboureihan St, Enghelab Ave, Tehran, Iran

## Abstract

**Background:**

Biomedical researchers have long looked for ways to diagnose and treat cancer patients at the early stages through biomarkers. Although conventional techniques are routinely applied in the detection of biomarkers, attitudes towards using Real-Time PCR techniques in detection of many biomarkers are increasing. Normalization of quantitative Real-Time PCR is necessary to validate non-biological alteration occurring during the steps of RNA quantification. Selection of variably expressed housekeeping genes (HKs) will affect the validity of the data. The aim of the present study was to identify uniformly expressed housekeeping genes in order to use in the breast cancer gene expression studies. Urokinase Plasminogen Activator was used as a gene of interest.

**Findings:**

The expression of six HKs (TFRC, GUSB, GAPDH, ACTB, HPRT1 and RPLP0) was investigated using geNorm and NormFinder softwares in forty breast tumor, four normal and eight adjacent tissues. RPLP0 and GAPDH revealed maximum M value, while TFRC demonstrated lowest M value.

**Conclusions:**

In the present study the most and the least stable genes were TFRC and RPLP0 respectively. TFRC and ACTB were verified as the best combination of two genes for breast cancer quantification. The result of this study shows that in each gene expression analysis HKs selection should be done based on experiment conditions.

## Introduction

Worldwide, breast cancer is the most frequent cancer among women. It affects more than one million women globally, accounting for more than 400,000 deaths annually [1]. In Iran as an Asian country, among women over 30, the incidence and prevalence rate of breast cancer is 22 and 120 per 100,000, respectively [2].

For a long time, biomedical scientists have been interested in finding ways to diagnose and to treat cancer patients at early stages. Research aimed at developing robust biomarkers and reliable assays, has made progress in the detection, diagnosis, and treatment of breast cancer. Only a limited number of biomarkers for breast cancer are currently available which assist in making breast cancer management decisions. Oncotype DX; a diagnostic panel commercially available; is indicated for specified breast cancer patients that predicts the risk of a patient experiencing a recurrence [3]. Estrogen receptor (ER), progesterone receptor (PR) and Her2/neu status are routinely measured to decide about hormone and targeted therapy. In addition, recommendations concerning the role of urokinase plasminogen activator (uPA) in detecting the invasive nature of the tumors have been recently added to the clinical guidelines [4].

Current routine assays for quantifying biomarkers, such as immunohistochemistry (IHC) and Enzyme-linked immunosorbent assay (ELISA) are approved and valid, and are reproducible between different laboratories [5-8]. However, unquantifiable results and long procedure time are among the drawbacks of these methods which have persuaded researchers to seek alternative modern molecular based techniques such as quantitative Real-Time PCR (Q-RT-PCR) [9,10]. Q-RT-PCR is highly cost-effective, very fast, and one of the most sensitive and specific quantification methods for gene expression analysis [11,12].

To be approved as a routine alternative method for conventional techniques; however, a number of validations are necessary for Q-RT-PCR including HK validation. Differences occurring during the steps of RNA quantification would be normalized by endogenous controls (ECs). There are large scale gene expression profiling studies in which hundreds of HKs were identified [13-15]. During the years of using Q-RT-PCR, based on this belief that housekeeping genes have uniform expression regardless of biological conditions, they have been applied for quantification. Several studies have revealed significant alterations in the expression of a number of ECs affecting validity of expression analysis [16-20]. Since reliability of ECs affects the accuracy of the normalized data, reference gene selection plays an important role in this matter. HK validations have been performed for a number of genes but, to the best of our knowledge, no report has been found for uPA in breast cancer. The uPA is involved in various biological processes. Along with some other genes, elevated expression of uPA in plasminogen activation system takes part in tumor cell invasion and metastatic process. High level expression of this marker represents an unfavorable prognostic factor for metastasis in breast cancer [6,21]. The aim of the present study was to validate reference genes in order to select the most appropriate ECs for uPA quantification in breast cancer tissues.

## Methods

Breast tumor tissues (n = 40), normal tissues (n = 4) and normal adjacent tissues (n = 8), were taken from Iranian Center for Breast Cancer Biobank (ICBC-BB). According to the protocols followed by ICBC-BB, immediately after excisional biopsy or surgery, sample tissues were snap-frozen in liquid nitrogen and stored at -70°C. ICBC-BB is obliged to ethical guidelines and recommendations for biobanks on the storage and use of human biological samples. Clinical and histopathological features of patients are summarized in table 1.

**Table 1:** Clinical and histological data of malignant and normal adjacent breast tissues.

Tissue type	Age	Size (mm)	Tumor stage	grade	Histological type	Menoposal status	ER	PR	Her2/neu	P53
Adjacent	50									
Adjacent	38						-	-	-	+
Malignant	38	18	IIA	III	IDC	pre	+	+	-	
Malignant	38	20		III	IDC	pre	3+	3+	2+	
Malignant	58	35	IIIA	II	IDC	post	-	-	-	+
Malignant	80	10		I	IDC	post	+	+	-	
Malignant	54	30	IIB	II	IDC	post				
Adjacent							+	+	3+	+
Malignant	40	25	IIB	II	IDC	pre	+	+	-	+
Malignant	35	20		III	IDC	post				
Adjacent										
Malignant							+	+	-	+
Malignant	52	21	IIA	II	IDC	post	+	+	-	+
Malignant			III		IDC		+	-	-	+
Malignant	82	50	IIB	III	IDC	post				
Adjacent	43						+	+	-	-
Malignant	50	50	IIA	II	IDC	pre	3+	3+	-	
Malignant	44	20	IIIA	III	IDC	pre	+	+	-	
Malignant	51	20	IIA	II	IDC	post				
Malignant							+	+	-	-
Malignant	37	60	IIIB	II	IDC	post				
Malignant	45				IDC	pre	+	+	-	
Malignant	40	15	IB	II	IDC	pre	-	-	-	+
Malignant	42	20	IV	III	IDC	pre	+	+	-	+
Malignant	52	21	IIA	II	IDC	post	+	+	-	-
Malignant	50	8	IIA	II	IDC	post	+	+	-	
Malignant	54	30	IIA	II	IDC	post	-	-	-	-
Malignant	45		IV		IDC	pre				
Adjacent	45									
Adjacent	40									
Adjacent	52									
Adjacent	42						-	-	-	-
Malignant	53				IDC+DCIS	pre	+	+	3+	-
Malignant	34	10	IIB	II	IDC+DCIS	pre	-	-	-	+
Malignant	56	38	IIB	II	IDC	post	+	+	-	
Malignant	71	20	IV	II	IDC	post	-	-	-	-
Malignant	34	30	IIA	II	IDC	pre	+	+	3+	
Malignant	37			II	IDC	pre	-	-	-	
Malignant	48	30	IIB		ILC	pre	+	+	-	+
Malignant	43	18	I			pre	-	+	-	
Malignant	45	20	II	II	ILC	pre	-	-	3+	+
Malignant	39	100	IIIA	II	IDC	pre				
Malignant	34	60		III	IDC	pre				
Malignant										
Normal	25									
Normal	38									
Normal	32									
Normal							+	+	-	-
Malignant	50	15	IB	I	IDC	pre	-	-	3+	+
Malignant	32	10	IV	II	IDC	pre	+	-	-	
Malignant	60	30	IV	II	IDC	post	+	+	-	-
Malignant	41	50	IV	I	IDC	pre				

Stage grouping are based on American Joint Committee on Cancer (AJCC). Estrogen receptor(ER), progesterone receptor (PR), Her2/neu and P53 status are based on IHC results. Invasive ductal carcinoma (IDC), invasive lobular carcinoma (ILC)

Tissues (8-20 mg) were excised on dry ice and homogenized in 1 ml of RnxPlus (Cinnagen, Iran) to extract RNA according to manufacturer directions. Extracted RNAs were quantified by spectrophotometer (Hitachi, U-0080D, Japan) and the Absorbance ratio at 260/280 and 260/230 were measured to control the purity of the RNA. The integrity of RNA was confirmed by checking ribosomal RNA with electrophoresis on a 1% agarose gel. Then, 3.6 μg of RNA was treated with 18 unit of RNase-free DNase (Fermentas AB, Vilnius, Lithuania), 20 units of RNase inhibitor (Fermentas AB, Vilnius, Lithuania) and 2.4 μl of 25 mM MgCl2. The total volume of reaction was 30 μl. The reaction was incubated in 37°C for 15 min and then 90°C for 5 min to inactivate the DNase.1 μg of total RNA was transcribed to cDNA using Precision™reverse transcription kit (PrimerDesign Ltd, UK).

Six common housekeeping genes in breast cancer; TFRC, GUSB, GAPDH, ACTB, HPRT1 and RPLP0; were selected and their stability were examined in order to normalize expression of uPA. All primers and probes were designed using Gene Runner v.3.05 and confirmed with primer express 3.0 (Table 2). Amplification efficiency for each primer was approximated using 10 fold cDNA serial dilutions and calculated using 7500 software system ver. 2.0. The serial dilutions were performed using pooled cDNA from 15 tumor cDNAs with equal proportion. CDNA synthesis was done as mentioned above.

**Table 2:** Primer probe sets.

Target	Accession No.	Sequence	Melting TM	efficiency
GAPDH	NM_001002	F GAAGGTGAAGGTCGGAGTC	61.3	94
		R GGGTGGAATCATATTGGAACA	63.2	
		P ATTTGGTCGTATTGGGCGCCTGGT	74.9	
TFRC	NM_003234	F ACCGGCACCATCAAGCT	64.5	94
		R TGATCACGCCAGACTTTGC	65.2	
		P TGAAAATTCATATGTCCCTCGTGAGGCT	72.1	
RPLP0	NM_001002	F CGGACGAGGATATGGGATTTG	67.2	89
		R AGAAGTAAGCCTTTATTTCCTTGTTT	64.7	
		P TCACCAAAAAGCAACCAACTTAGCCAGT	72	
GUSB	NM_000181	F GCGTTCCTTTTGCGAGGAGA	68.6	74
		R GGTGGTATCAGTCTTGCTCAA	64.7	
		P ACCAGGTATCCCCACTCAGTAGCCAAG	72	
HPRT1	NM_000194	F TGGACTAATTATGGACAGGACTGAA	64.4	103
		R GTAATCCAGCAGGTCAGCAA	62.8	
		P CTTGCTCGAGATGTGATGAAGGAGATGG	73.7	
UPA	NM_002658	F AGGGCAGCACTGTGAAATAGATAAGT	65.7	97
		R CATGGTACGTTTGCTGAAGGA	64.8	
		P TTACCGAGGAAAGGCCAGCACTGACA	75.3	
ACTB	NM_001101	F CAGCAGATGTGGATCAGCAAG	65.9	95
		R GCATTTGCGGTGGACGAT	67.1	
		P AGGAGTATGACGAGTCCGGCCCC	73.8	

Probes were labeled with 5' FAM and 3' TAMRA.

### Quantitative Real-Time PCR

Q-RT-PCR was carried out in triplicate format within the same 96-well plate for each sample using precision™ 2 × qPCR Mastermix (PrimerDesign Ltd, UK) in 20 μl reactions. Primer and probe concentrations were 0.5 μM and 0.3 μM, respectively. Fluorescent detection was performed using Applied Biosystems 7500 System. Data were analyzed using SDS software, vers.2.0 (Applied Biosystems).

### Data analysis and endogenous control stability

Raw data of Q-RT-PCR was analyzed using SDS software, vers.2.0 (Applied Biosystems). Samples with standard deviation greater than 0.5 from the mean threshold cycle (CT) of the replicates were excluded. In order to find the most stable housekeeping gene, the data were transformed to linear scale values with Excel. Analyses were done by pairwise comparison approach applying geNorm software[19] as well as combined estimation of the intra and inter group validation using NormFinder software[22]. UPA expression measurement was performed using ΔΔCT method with RPLP0 or combination of ACTB and TFRC as endogenous control. Comparison between the mean of each group was done using paired sample t-test (SPSS v.13) with 95% confidence interval.

## Results

Threshold cycle (CT) values of endogenous controls and uPA are shown in table 3.

**Table 3:** threshold cycle (CT) values of endogenous controls and uPA.

Gene	CT Range	CT Min	CT Max	Mean ± s.e.m
RPLP0	22.68	19.52	42.2	29.69 ± 0.81
GUSB	8.61	27.59	36.2	31.99 ± 0.33
TFRC	15.64	25.27	40.92	31.7 ± 0.5
HPRT1	14.25	26.42	40.67	32.61 ± 0.43
ACTB	14.05	16.93	30.98	25.1 ± 0.5
GAPDH	19.2	22.69	41.89	29.3 ± 0.5
UPA	10.65	28.13	38.78	31 ± 0.38

The range of threshold cycles (CT Range) was between 8.61 and 22.68 among endogenous candidate genes with a mean value ranging from 25.1 (± 0.5 s.e.m) for ACTB to 32.61(± 0.43 s.e.m) for GUSB. The maximum and minimum expression ranges were 22.68 cycles for GUSB and 8.61 cycles for ACTB respectively (Table3).

**Figure 1:**
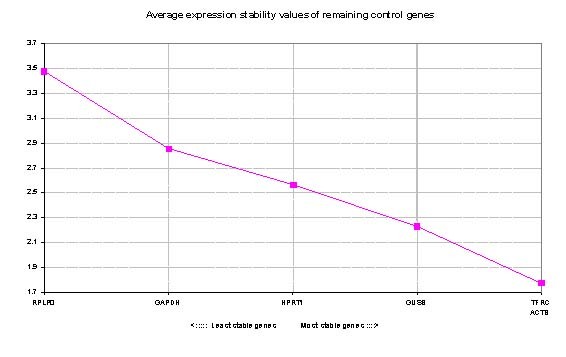
**GeNorm analysis of candidate genes: genes with low Average expression stability M which are plotted in the right side of x-axis indicate greater stability**.

**Figure 2:**
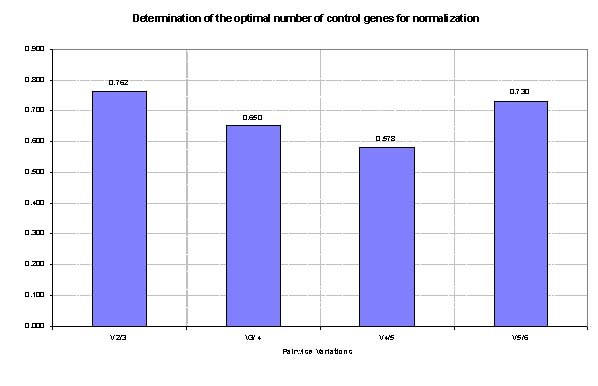
**Determination of the optimal number of ECs for normalization: Inclusion of additional reference gene is not required for pairwise variation values (V value) below 0.15**.

Data were analyzed using geNorm and NormFinder software programs. GeNorm calculates pairwise variation to find the most stable expressed ECs, and thereby computing the average expression stability value (M) and plotting it for each gene. In this study, RPLP0 and GAPDH revealed maximum M while TFRC demonstrated lowest M value (Figure 1). The most stable expressed gene was TFRC. The GeNorm software also calculates normalization factor (V); the point at which addition of extra endogenous control is unnecessary; to find the optimal number of required ECs. According to GeNorm software manual, recommended V-value cut-off was 0.15 but it was also mentioned that proposed value must not be taken as a too strict cut-off. In this study the V-values of using 3 and 4 ECs were calculated as 0.5 and 0.4, respectively (Figure 2).

NormFinder software; an algorithm for identifying the optimal normalization gene among a set of candidates; was used to rank the set of candidate normalization genes according to their expression stability in a given sample set. In this study, NormFinder determined TFRC as the most stable EC Followed by GUSB and ACTB genes (Table 4).

**Table 4:** Stability values of reference genes using geNorm and NormFinder programs

	geNorm		NormFinder	
**Rank**	**Gene name**	**Stability (M value)**	**Gene name**	**Stability**

1	TFRC	2.846	TFRC	0.796
2	GUSB	2.902	GUSB	1.007
3	ACTB	2.984	ACTB	1.130
4	HPRT1	3.298	HPRT1	1.799
5	GAPDH	3.538	GAPDH	1.984
6	RPLP0	4.737	RPLP0	2.966

Genes with lower number are more stable.

Statistical comparison of "uPA expression results normalized with RPLP0" in one group and "the combination of TFRC and ACTB" in another group showed significant difference in uPA expression amounts (p-value < 0.05).

## Discussion

During recent years traditional attitudes towards therapeutic care has been replaced with individualized medicine especially in the cancer field. It is recommended to assess approved markers in order to select the best therapeutic models instead of blind administration of similar regimens for all patients.

Among current systems, gene expression profiling plays a major role in tailoring treatment to the individual strategy. Despite the accuracy of routinely used approved methods for detection of biomarkers such as IHC for measurement of Her2 and ER biomarkers and ELISA for uPA, there are still disadvantages. For instance, ELISA requires a substantial amount of tissue and small tissues of early stage cancer would be difficult to be analyzed [4]. Several studies using various types of design have been conducted to determine whether novel molecular techniques like Q-RT-PCR may be added to routine approved methods for detection and quantification of biomarkers [23-31].

To our knowledge this is the first study on determining reference gene in breast cancer for quantification of uPA. We found that TFRC and ACTB is the best combination of two genes with the greatest expression stability. GeNorm and NormFinder without sub grouping had a similar performance in detecting the most and the least stable genes. TFRC and RPLP0 were the most and the least satiable genes. GAPDH and ACTB were among the least and the most stable genes which a in concordance with other breast cancer studies [17,32-35]. Both softwares recognized the same order of stability for all of the genes (Table4). Other Studies suggest different genes. Mc Neill et al in a study for ER quantification, suggested MRPL19 and PPIA as the most stable and RPLP0 as the least stable genes, while in the study by Lyng et al TBP, RPLP0 and PUM1 were recommend for normalization [36]. Moreover, 18S rRNA and HPRT1 have been suggested for breast cancer normalization in quantification of Her2/neu [18,34].

Housekeeping selection is a critical step in Q-RT-PCR analysis. The idea that housekeeping genes (ECs) pose constant expression in different cells may influence their selection in various studies. In the study by Mc Neill et al the data of housekeeping gene selection in colorectal and ovarian cancer was used to decide about selection of an EC for breast cancer [17]. It should be noted that instability of one housekeeping gene in specific cancer does not mean instability in other cancers. The important key is that housekeeping gene expression patterns are influenced by cancer mechanism and EC selections should be made for each cancer separately [37,38]. Housekeeping generalization may result in inappropriate set of ECs because some genes may be included or excluded erroneously based on other cancer evidences.

It is also noteworthy that various experiment conditions in the studies may change the expression of the housekeeping genes. As a result, dissimilar genes may be found as the best reference for normalization in different studies with different conditions.

## Conclusions

To conclude, it appears that identifying a *universal housekeeping *for gene expression analysis is far from reality. Thus, stability of controls should be checked based on the tissue type and experiment design. On the basis of our findings, we suggest that TFRC is the most stable EC, ACTB and TFRC is the best combination of two reference genes to quantify uPA, and that using RPLP0 and GAPDH are not recommendable for breast cancer. The authors also suggest testing with large sample size and more candidate reference gene to find more stable ECs.

## Competing interests

The authors declare that they have no competing interests.

## Authors' contributions

KM conceived and designed the project and critically reviewed data analysis and manuscript. RE designed the experiment, was responsible for experiments set up, data analysis and writing the manuscript. NA was responsible for collecting samples and performing the experiments. All authors read and approved the final manuscript.
